# The Role of Nitric Oxide in Nitrogen Fixation by Legumes

**DOI:** 10.3389/fpls.2020.00521

**Published:** 2020-06-03

**Authors:** Santiago Signorelli, Martha Sainz, Sofía Tabares-da Rosa, Jorge Monza

**Affiliations:** ^1^Laboratorio de Bioquímica, Departamento de Biología Vegetal, Facultad de Agronomía, Universidad de la República, Montevideo, Uruguay; ^2^The School of Molecular Sciences, Faculty of Science, The University of Western Australia, Crawley, WA, Australia; ^3^Australian Research Council Centre of Excellence in Plant Energy Biology, University of Western Australia, Crawley, WA, Australia

**Keywords:** ⋅NO, reactive oxygen species, leghemoglobin, legumes, nitrogen fixation, reactive nitrogen species

## Abstract

The legume-rhizobia symbiosis is an important process in agriculture because it allows the biological nitrogen fixation (BNF) which contributes to increasing the levels of nitrogen in the soil. Nitric oxide (⋅NO) is a small free radical molecule having diverse signaling roles in plants. Here we present and discuss evidence showing the role of ⋅NO during different stages of the legume-rhizobia interaction such as recognition, infection, nodule development, and nodule senescence. Although the mechanisms by which ⋅NO modulates this interaction are not fully understood, we discuss potential mechanisms including its interaction with cytokinin, auxin, and abscisic acid signaling pathways. In matures nodules, a more active metabolism of ⋅NO has been reported and both the plant and rhizobia participate in ⋅NO production and scavenging. Although ⋅NO has been shown to induce the expression of genes coding for NITROGENASE, controlling the levels of ⋅NO in mature nodules seems to be crucial as ⋅NO was shown to be a potent inhibitor of NITROGENASE activity, to induce nodule senescence, and reduce nitrogen assimilation. In this sense, LEGHEMOGLOBINS (Lbs) were shown to play an important role in the scavenging of ⋅NO and reactive nitrogen species (RNS), potentially more relevant in senescent nodules. Even though ⋅NO can reduce NITROGENASE activity, most reports have linked ⋅NO to positive effects on BNF. This can relate mainly to the regulation of the spatiotemporal distribution of ⋅NO which favors some effects over others. Another plausible explanation for this observation is that the negative effect of ⋅NO requires its direct interaction with NITROGENASE, whereas the positive effect of ⋅NO is related to its signaling function, which results in an amplifier effect. In the near future, it would be interesting to explore the role of environmental stress-induced ⋅NO in BNF.

## Introduction

The biological nitrogen fixation (BNF) is the process of reducing atmospheric nitrogen (N_2_) to ammonium (NH_4_^+^) catalyzed by the NITROGENASE enzyme. This process is carried out by a small group of bacteria, in either free-living condition, associated with different plants such as epiphytes or endophytes, or establishing endocellular symbiosis with legumes (Masson-Boivin et al., 2009). The rhizobium-legume symbiosis involves the exchange of carbon source produced by the plant and ammonium fixed by the bacteria in specialized organs denominated nodules. This symbiosis helps legumes to naturally colonize nitrogen-poor soils. Thus, this symbiosis positively impacts on agriculture, not only for the savings of nitrogen (N) fertilizers but also due to the reduction of its negative impact on the environment, which is key to achieve sustainable agriculture. Particularly because of the importance of sustainable agriculture, the interest in the BNF has been revitalized in recent years (Sulieman and Tran, 2015), with an emphasis in the rhizobium-legume symbiosis involving the cultivation of food and fodder (Lindström et al., 2010; Laranjo et al., 2014). Moreover, researchers have done considerable efforts to introduce the ability to perform BNF into non-leguminous plants, either by introducing the NITROGENASE enzyme into plants through genetic engineering (Oldroyd and Dixon, 2014) or by using *Gluconacetobacter diazotrophicus*, a non-nodulating endophytic nitrogen-fixing bacterium (Cocking et al., 2006).

Nitric oxide (⋅NO) is a small free radical molecule, which is ubiquitous in plants and its production is often enhanced under stress conditions (Corpas et al., 2008, 2011; Signorelli et al., 2013, 2019). ⋅NO acts as a signaling molecule interacting with hormone signaling in plants (Klessig et al., 2000; Wang et al., 2015a) and regulating different developmental processes such as germination, root elongation, floral transitions, branching, and ripening (Lozano-Juste and Leon, 2011; Sanz et al., 2014; Chaki et al., 2015). The BNF is also modulated by ⋅NO at different stages of this process. In this review, we present and discuss the metabolism of ⋅NO in nodules, the different sources of ⋅NO, and its effect on nodule establishment, BNF and nodule senescence. With this, we attempt to provide clear views on what is currently know and highlight the outstanding questions that need to be investigated in this exciting research area.

## ⋅NO in Legume-Bacteria Interaction

The establishment of the legume-rhizobium symbiosis requires the recognition between the rhizobia and legume and the formation of nodules, the plant organ hosting the rhizobia and where the BNF takes place. The nodule provides a low oxygen (microoxic) environment which is necessary to prevent the inhibition of the NITROGENASE activity by oxygen (O_2_). To cope with the lower O_2_ availability, the cytochrome-pathway of respiration of mitochondria from nodules has a higher apparent affinity for O_2_ than the equivalent of mitochondria from roots (Millar et al., 1995). This allows the nodule cells to produce ATP by oxidative phosphorylation which is used for the rhizobia as the energy source, together with other carbon sources, to fixate nitrogen. The fixated nitrogen is assimilated mostly in the cytosol of the nodule cells and taken up by the plant.

**FIGURE 1:**
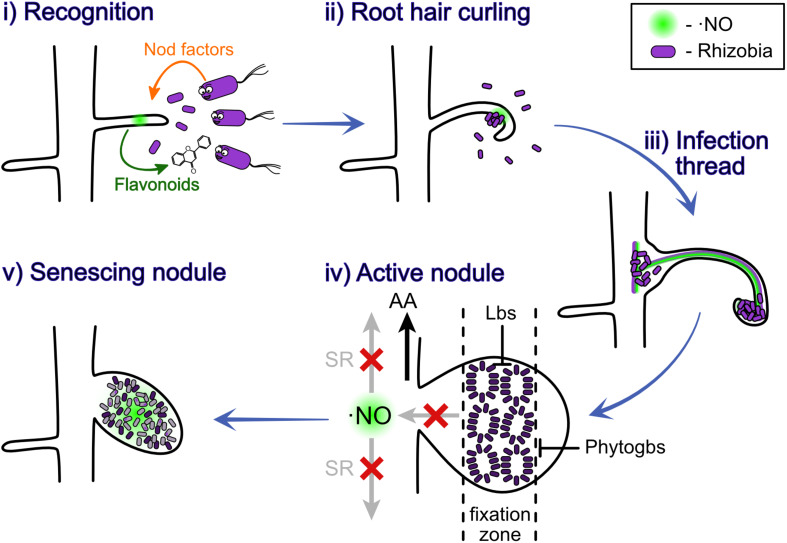
Nitric oxide participates at different stages of the nodulation process. ⋅NO was observed to be involved at all the different stages of nodulation, plant-rhizobium recognition (i), root hair curling (ii), infection thread (iii), nitrogen fixation in active nodules (iv), and senescing nodules (v). The light purple indicates the bacteria; the dark purple indicates the bacteroids; the gray indicates dead bacteroids; and the diffuse light green indicates ⋅NO. AA, amino acids; Phytogbs, PHYTOGLOBINS; Lbs, LEGHEMOGLOBINS; SR, systemic response.

Two genetic pathways were shown to control the number of nodules produced in the root nodule symbiosis, one of them controlling the rhizobial infection and the other one controlling the nodule organogenesis (Penmetsa et al., 2003). Depending on the legume, the nodule can be indeterminate or determinate, which means respectively that they sustain or not meristematic activity (Hirsch, 1992). The nodulation process starts with the release of chemical signals by the root hairs, which attract rhizobia and trigger them to produce bacterial nodulation factors (Nod-factors) (Figure 1 i). These Nod factors are perceived by the legume to promote root cell division and other downstream responses (Geurts and Bisseling, 2002). The rhizobia grow, induce a curl in the root hair (Figure 1 ii) and a tubular and intracellular structure containing the bacteria, known as infection thread, is formed (Figure 1 iii). As the rhizobia reproduce, the infection thread grows reaching first the base of root hair cell and later the nodule primordium (Geurts and Bisseling, 2002). The formation of this infection threat it also induced by the presence of the plant hormones cytokinin (CK) and auxin (AUX) (Roy et al., 2020). Afterward, the infection thread starts to release bacterial cells into the parenchyma cells of the developing nodule, where the bacterial cells differentiate into bacteroids, a transformation that requires morphological and physiological changes (Figure 1 iv). For a detailed review of the nodule formation process, we recommend revising Gage (2004) or Roy et al. (2020).

In the different stages of the nodule formation, a crosstalk between the bacteria and the root cells occurs. Reactive oxygen species (ROS) are present among the molecules involved in this communication. ROS are produced at a high level in the nodule, mainly due to the high demand of respiration to support NITROGENASE activity and the autoxidation of oxygenated leghemoglobin (oxy-Lb) (Puppo et al., 1981). Therefore, the role of ROS on modulating the legume-rhizobia interaction, as well as the relevance of the antioxidant system to control them have been widely studied (Becana et al., 2010). Likewise, the presence of ⋅NO in the nodule is well documented (Baudouin et al., 2006; Nagata et al., 2008; del Giudice et al., 2011; Cam et al., 2012; Fukudome et al., 2016, 2018; Calvo-Begueria et al., 2018). Due to its reactive nature, ⋅NO and other reactive nitrogen species (RNS) can also interfere with this plant-rhizobia communication.

In the specific interactions *Lotus japonicus-Mesorhizobium loti* and *Medicago sativa-Ensifer meliloti*, ⋅NO was observed to be induced in the roots of plants 4 h post-inoculation (hpi), suggesting that ⋅NO participates at early stages of the plant-rhizobia interaction (Nagata et al., 2008). Interestingly, ⋅NO was only perceived at 4 hpi but not at 10 and 24 hpi, and this effect was not observed when the roots were inoculated with unspecific rhizobium (Nagata et al., 2008). In contrast, *L. japonicus* showed a higher and sustained accumulation of ⋅NO in roots when infected with different plant pathogens (Nagata et al., 2008). These observations suggest that the peak response observed at 4 hpi is a signal-recognition response, rather than a stress response. Different kinetics for ⋅NO accumulation were observed in the *Medicago truncatula–E. meliloti* symbiosis, but the results were consistent in the fact that ⋅NO is induced and necessary for the correct establishment of the symbiosis (del Giudice et al., 2011). In this symbiosis, the induction of ⋅NO was detected 2 days post-infection (dpi), during the curling of the root hair, and 6 dpi, when the infection thread is formed (del Giudice et al., 2011). Using reporter bacteria responsive to ⋅NO, the authors showed that the bacteria respond to the endogenously produced ⋅NO in the infection pocket, and when ⋅NO is specifically scavenged, the development of the nodule is delayed (del Giudice et al., 2011). Together, this evidence demonstrates that ⋅NO promotes the legume-bacteria interaction and nodule development, potentially acting as a signaling molecule (discussed in section “Signaling Role of ⋅NO in Nitrogen Fixation”).

At later stages of this symbiosis ⋅NO was also shown to be present in *M. truncatula* nodules, both in developing (10 dpi) and mature nodules (30 dpi), in particular, in the fixation zone (Baudouin et al., 2006). Furthermore, ⋅NO was observed to locally induce nodule senescence in *M. truncatula* (Cam et al., 2012) and *L. japonicus* (Fukudome et al., 2018). These findings suggest the involvement of ⋅NO also during nitrogen fixation and nodule senescence (Figure 1).

Taking all together, the current evidence clearly shows the involvement of ⋅NO during the legume-rhizobia interaction. In many cases, a positive effect of ⋅NO on this interaction is observed, although the mechanism is not fully understood (discussed in section “Perspectives”).

## Metabolism of ⋅NO in Nodules

Plants can produce ⋅NO by different ways, some of them non-enzymatic, as the non-enzymatic reduction of nitrite (Bethke et al., 2004), and others are dependent on enzymes such as the NITRATE REDUCTASE (NR), PLASMA MEMBRANE-BOUND NITRITE: ⋅NO REDUCTASE, mitochondrial-electron transport chain-dependent (mETC-dependent) nitrite-reducing activity, and the NOS-like activity (Stöhr et al., 2001; Planchet et al., 2005; Corpas et al., 2009; Astier et al., 2018). POLYAMINE OXIDASES and HYDROXYLAMINE OXIDASE have been also suggested to contribute to ⋅NO production (Tun et al., 2006; Rümer et al., 2009). Despite the several mechanisms involved in ⋅NO production, the NR and mETC-dependent reduction of nitrite are the better understood mechanisms to contribute to ⋅NO production, being the latter only relevant under microoxic and anoxic conditions (León and Costa-Broseta, 2020). Interestingly, under the nodulation process, the three genes encoding for NR of *M. truncatula* were shown to be induced (Damiani et al., 2016). In fact, the authors suggested a specific role of these enzymes as ⋅NO source in nodulation. Nonetheless, recent experiments coupling EPR (electron paramagnetic resonance) and DAF-2 (4,5-diaminofluorescein) to detect ⋅NO in bean and soybean nodules suggested that ⋅NO is also produced by nitrate- and arginine-independent pathways (Calvo-Begueria et al., 2018).

**FIGURE 2:**
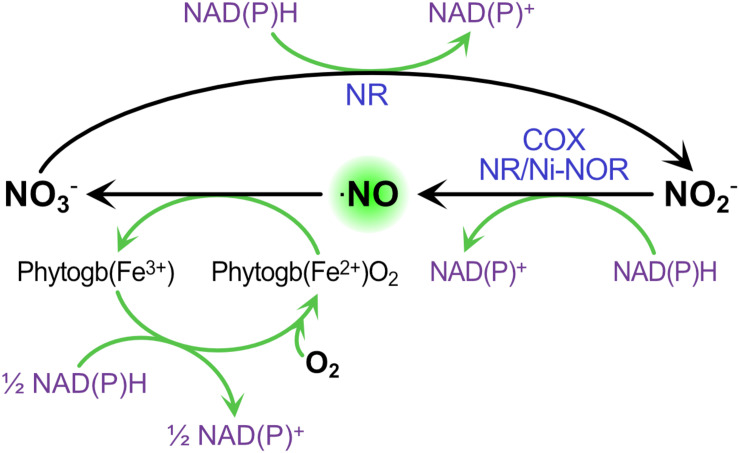
The contribution of Phytogb/⋅NO cycle to ⋅NO production and NAD(P)H re-oxidation during hypoxia. ⋅NO is oxidized to nitrate by OXYPHYTOGLOBIN [Phytogb(Fe_2_^+^)O_2_], which turns to METPHYTOGLOBIN [Phytogb(Fe^3+^)]. Nitrate is then reduced to nitrite through NITRATE REDUCTASE (NR), and nitrite is reduced to ⋅NO though NR, the PLASMA MEMBRANE NITRITE-⋅NO REDUCATSE (NI-NOR) or the CYTOCHROME OXIDASE (COX). NAD(P)H oxidation occurs in the reactions forming ⋅NO and its subsequent oxidation back to nitrate.

Under microoxic conditions, it is well known that nitrite can be used as final electron acceptor of the mETC to produce ⋅NO (Horchani et al., 2010; Gupta and Igamberdiev, 2011). Therefore, once the nodule is established, the microoxic condition is generated within the nodule promoting this extra source of ⋅NO. In this situation, ⋅NO is involved in the cycle named Phytoglobin-⋅NO (Phytogb-⋅NO), in which the ⋅NO produced from nitrite by the mETC diffuses into the cytosol where it is oxidized into nitrate by PHYTOGLOBINS (Phytogbs), and the resulting nitrate is reduced back to nitrite by NR and transported to the mitochondria where the cycle is repeated (Figure 2; Stoimenova et al., 2007; Igamberdiev et al., 2010; Gupta and Igamberdiev, 2011). This Phytogb-⋅NO cycle was suggested to function as an alternative mechanism to the classic fermentation pathways (ethanol and lactate formation) for the re-oxidation of NAD(P)H to NAD(P)^+^ during hypoxia (Igamberdiev and Hill, 2004). In microoxic cells, the energy production for short-term viability is achieved mainly through glycolysis. The Phytogb-⋅NO cycle would allow NADH re-oxidation during the reduction of NO_3_^–^ to ⋅NO_2_^–^ and in the regeneration of reduced (Fe^2+^) Phytogb (Figure 2), being the resulting NAD^+^ available for the glycolytic process (Igamberdiev and Hill, 2004).

Besides the electron transport chain of the nodule cells, the electron transport chain of some rhizobia also contributes to ⋅NO production (Horchani et al., 2010) through the denitrification pathway, being the highest contribution for ⋅NO biosynthesis from the bacteroid side (Hichri et al., 2015). In the *Bradyrhizobium* genera, different strains have been shown to be able to denitrify under microoxic conditions, both in free-living conditions (Monza et al., 2006) and in symbiosis (Bedmar et al., 2005). Under flooding conditions however, in soybean nodules, the periplasmic NR and NiR of *Bradyrhizobium diazoefficiens* (also known as *Bradyrhizobium japonicum*) were suggested to be the main sources of ⋅NO (Sánchez et al., 2010). Although the contribution of ⋅NO by the bacteria is considered to be lower than that by the plants (Baudouin et al., 2006; Horchani et al., 2010), both partners are involved in the production of this molecule in mature nodules. Therefore, it is not surprising that some genes involved in both ⋅NO production and degradation have proven to be more expressed in mature nodule when compared to developing nodule (Berger et al., 2019). This indeed suggests that a more active metabolism of ⋅NO takes place in established nodules.

Finally, it is important to remark that ⋅NO is highly permeable to biological membranes (permeability coefficient in the membrane of 93 cm ⋅ s^–1^, Signorelli et al., 2011), meaning that ⋅NO can diffuse in and out the bacteroids. Thus, the lack of a unique ⋅NO source together with its capacity to diffuse between the plant and the rhizobia set a complex scenario to elucidate the main source of ⋅NO in this interaction.

### *S*-Nitrosothiols In Nodule

Nitric oxide can modulate the activity of different enzymes mainly through *S*-nitrosylation (also known as *S*-nitrosation). This reversible posttranslational modification of proteins is the covalent binding of a ⋅NO group to the thiol group of a cysteine residue leading to the formation of an *S*-nitrosothiol (SNO). Different mechanisms have been shown to mediate this modification, varying on the state of the cysteine (as radical or anion) and the nature of the ⋅NO group (as radical, anion, cation or transiently associated to other molecules) (Kovacs and Lindermayr, 2013). This modification produces structural changes in the protein, and when the modified residues are close enough to the active site, its activity is affected. The major effects of ⋅NO on biological processes are due to its capacity to induce post-translational modifications of key proteins involved in signaling cascade pathways, resulting in the up or down-regulation of downstream components such as transcription factors, which in turn affect the expression of a plethora of genes. For instance, ⋅NO is known to inactivate several proteins involved in the ABA signaling pathway that controls the activity of the transcription factor ABI5, resulting in the suppression of the ABA-mediated responses (Signorelli and Considine, 2018). Likewise, ⋅NO can target the group VII of ETHYLENE RESPONSE FACTORS (ERF) to proteasomal degradation (Gibbs et al., 2014), affecting the regulation of different process under regulation of these transcription factors, such as abiotic/biotic stress responses and developmental processes (Gibbs et al., 2015; Considine et al., 2017).

The inhibition of NITROGENASE by ⋅NO has been known since long time (Trinchant and Rigaud, 1982; Kato et al., 2010). Although this inhibition can be through the formation of metal-nitrosyl complex (Michalski and Nicholas, 1987), in the symbiosis *M. truncatula-E. meliloti* two NITROGENASE proteins (encoded by *nifK* and *nifH*) were reported to be *S*-nitrosylated (Puppo et al., 2013), which could present an alternative mechanism to post-translationally regulate the NITROGENASE activity in the bacteroids. In the same symbiosis, many proteins related to TCA cycle and carbohydrate metabolism were reported to be *S*-nitrosylated in both the legume and the partner rhizobium (Puppo et al., 2013). Moreover, in this legume three proteins involved in amino acid metabolism, the ASPARAGINE SYNTHETASE, GLUTAMINE SYNTHETASE, and S-ADENOSYLMETHIONINE SYNTHETASE were also reported as *S*-nitrosylated (Melo et al., 2011; Puppo et al., 2013). Although there have been some proteins identified as *S*-nitrosylated, in many cases the consequence of such modification on the enzymatic activity is still unknown. Conversely, the GLUTATHIONE PEROXIDASE 1 (GPX1) from nodules of *M. truncatula* is known to be reversibly inactivated by *S*-nitrosylation (Castella et al., 2017). As this enzyme participates in the transmission of redox signals, it was suggested that ⋅NO can exert part of its signaling function through the modulation of this protein (Castella et al., 2017).

Nevertheless, it is important to remark that ⋅NO levels do not always correlate with SNOs levels. This was clearly observed in non-nodulated roots of the model legume *L. japonicus* (Signorelli et al., 2019) and in nodules of *Arachis hypogaea* (Maiti et al., 2012). Thus, low levels of ⋅NO do not necessarily implicate that *S*-nitrosylation will not take place. Moreover, in *A. hypogaea* nodules it was observed that the levels of cytoplasmic SNOs were lower in the nodule than in the symbiotic bacteria; thus the authors suggested that the bacteria contribute to the protection of *S*-nitrosylation in the nodule and that this interaction might implicate the transfer of redox compounds between the bacteroids and the nodule cells (Maiti et al., 2012). Also, the number of SNOs in the nodule was observed to increase with the age of the nodule (Maiti et al., 2012).

Interestingly, a phytogb (AHb1) from *Arabidopsis thaliana* was the first protein of plants reported to be *S*-nitrosylated and the authors suggested this could be a mechanism to eliminate ⋅NO during hypoxic stress (Perazzolli et al., 2004). The authors also tested if the overexpression of AHb1 reduced the ⋅NO-mediated hypersensitive cell death in response to pathogens, but it was not the case (Perazzolli et al., 2004). Conversely, the detoxification of SNOs was proven to increase disease resistance upon infection with *Pseudomonas syringae* (Feechan et al., 2005). Therefore, increased SNO levels are believed to enhance the susceptibility to pathogens and change the redox status of the cells resulting in the activation of antioxidant responses (Begara-Morales et al., 2019 and references therein). In this sense, it would be interesting to understand if changes in SNOs, prior to rhizobium infection, promote or reduce the success of the infection. Understanding this could lead to the use of better management practices to promote rhizobia inoculation.

Together, the evidence demonstrates that SNOs occurs *in vivo* in legume nodules and, in some cases, the activity of the proteins is affected by this posttranslational modification. In the near future, it is expected that an increased number of *S*-nitrosylated proteins are going to be reported. For those proteins that have been already reported as *S*-nitrosylated, it would be important to understand the consequences on their activity.

## ROS and RNS Homeostasis in the Nodule

### ROS and RNS Are Induced in the Establishment of the Legume-Rhizobia Interaction

Reactive oxygen species are formed due to partial reductions of O_2_ and can react with cellular components leading to irreparable metabolic dysfunction or cell death. As ROS are ubiquitous in aerobic organisms, they tend to be controlled under normal conditions by different antioxidant systems. In such controlled conditions, ROS are essential for certain cellular functions. ROS include free radicals, such as superoxide radical (O_2_⋅^–^) and hydroxyl radical (⋅OH), and non-radical compounds like hydrogen peroxide (H_2_O_2_) and singlet oxygen (^1^O_2_). The term RNS is used to designate ⋅NO and related molecules such as peroxynitrite (ONOO^–^), nitrogen dioxide (⋅NO_2_), dinitrogen trioxide (N_2_O_3_) and SNOs. Like ROS, RNS can be moderately (e.g., ⋅NO, SNOs) or highly (e.g., ONOO^–^, ⋅NO_2_) reactive. RNS, directly or indirectly, participate in the post-translational modifications of proteins, which are involved in the cellular signaling process in both physiological and pathological conditions.

During the plant-rhizobium interaction, ROS play an important role and its production by the plants is known to be triggered, at least in part, by compatible Nod factors (Ramu et al., 2002). The silencing of the apoplastic-O_2_⋅^–^-producing enzyme, RESPIRATORY BURST OXIDASE HOMOLOG (RBOH), negatively affected the symbiotic nitrogen fixation in different legumes (Marino et al., 2011; Arthikala et al., 2017). The plants also respond producing a peroxidase (RIP1) as a mechanism to prevent the excessive generation of H_2_O_2_ (Ramu et al., 2002). Therefore, both the ROS generation and the induction of antioxidant system to control ROS levels are essential for the correct establishment of the symbiosis (Ramu et al., 2002; Becana et al., 2010). Legumes count with the common antioxidant enzymes such as SUPEROXIDE DISMUTASE, CATALASE, and diverse PEROXIDASES, and non-enzymatic antioxidants such as ascorbate and glutathione as the first barrier to protect against ROS. Here the general antioxidant machinery will not be discussed. Instead, we describe those systems specific from the legume-rhizobia symbiosis.

### Phytoglobins

Nodule function requires the protein LEGHEMOGLOBIN (Lb), which transport and deliver O_2_ to the symbiosomes at a low but steady concentration that allows efficient bacteroid respiration while preventing NITROGENASE inactivation (Appleby and Bergersen, 1980). Besides Lbs, plants possess many other HEMOGLOBINS (Hbs), currently known as Phytogbs, which function is less clear in plants but have been linked to the control of RNS homeostasis (Dordas et al., 2003; Perazzolli et al., 2004; Nagata et al., 2008). Here, we introduce the different plant Hbs and their potential role in controlling ROS and RNS.

In vascular plants, Phytogbs can be divided into six types based on phylogenetic analyses and biochemical properties (Hill et al., 2016). Phytogb0 are localized in any organ of gymnosperms but also in algae and bryophytes, and have moderate to high affinity for O_2_ (Garrocho-Villegas and Arredondo-Peter, 2008). Phytogbs1 have extremely high O_2_ affinities, making them unsuitable for O_2_ transport and delivery (Smagghe et al., 2009), and their main function seems to be related to the modulation of ⋅NO (Gupta et al., 2011b) and preserving cellular energy during hypoxia (Hill, 2012). In *L. japonicus*, the expression of *Ljphytogb1-1* gene increases in response to symbiosis with *M. loti*, but not when it interacts with pathogenic microorganisms so it has been proposed that Ljphytogb1-1 eliminates the ⋅NO produced in the initial response to the infection, allowing the establishment of symbiosis (Nagata et al., 2008). Very recently, it has been shown in *M. truncatula* that Mtphytogb1-1 regulates the concentration of ⋅NO both during early symbiosis steps and in mature nodules (Berger et al., 2020). Phytogbs2 display O_2_ affinities that resemble those of Lbs (Dordas, 2009), and their expression is known to be induced by CK treatments and cold conditions (Hunt et al., 2001). Vigeolas et al. (2011) proposed that this type of Phytogbs could facilitate the supply of O_2_ to developing tissues. Phytogbs1 and Phytogbs2 are localized in any organ of angiosperms. Phytogbs3 represent a group with very low similarity to Phytogbs1 and Phytogbs2 and have moderate O_2_ affinities (Watts et al., 2001). A biochemical property that defines Phytogbs3 is that they have a 2-on-2 α-helical sandwich secondary structure instead of the canonical 3-on-3 structure of other Phytogbs (Wittenberg et al., 2002). Although the functions of Phytogbs 3 are unknown, some of them are induced in nodules and mycorrhizal roots (Zhu et al., 2005) and might also interact with ⋅NO (Sanz-Luque et al., 2015). It has been suggested in *M. truncatula* that the function of two Phytogbs3 could be related to symbiosis by suppressing the initial defensive response of the plant due to the ability of these proteins to bind ⋅NO (Vieweg et al., 2005). In addition, some of their bacterial counterparts have been implicated in tolerance to nitrosative stress (Angelo et al., 2008). Both Lbs and SymPhytogb are proteins exclusively related to the symbiotic process facilitating a steady low O_2_ supply to the bacterial microsymbionts (Smagghe et al., 2009). They present moderate to high O_2_ affinity and are specifically localized in N_2_-fixing nodules of legumes and actinorhizal or any other non-legume plant, respectively.

### The Involvement of Plant Phytogbs in ROS and RNS Metabolism

In nodules, high respiration rates together with a high concentration of Lb and the abundance of catalytic Fe enhance, among other things, nodule capacity to generate ROS (Marino et al., 2009). Using leghemoglobin-RNA interference lines of *L. japonicus*, Günther et al. (2007) demonstrated that loss of Lb results in significantly lower H_2_O_2_ levels in nodules, which suggested a role of Lb in *in vivo* ROS production. Like other Hbs, oxy-Lb (Lb^2+^O_2_) auto-oxidizes spontaneously to form ferric (or meta) Lb (Lb^3+^) and O_2_⋅^–^, especially under the slightly acid pH of nodules (Puppo et al., 1981). The released O_2_⋅^–^ can, in turn, oxidize other oxy-Lb molecules to Lb^3+^, which enhances the inactivation of ferrous Lb (Lb^2+^) and oxy-Lb. Oxy-Lb and Lb^3+^ can also be oxidized by H_2_O_2_ (Puppo et al., 1982). The reaction between H_2_O_2_ and oxy-Lb or ferric Lb, in equimolar proportion, forms ferryl Lb (Lb^*IV*^), a stable but inactive form of Lb (Aviram et al., 1978; Sievers et al., 1978). When Lb and H_2_O_2_ are in a 1:2 ratio, radicals can be formed in the Tyr residues of the protein, which react forming 2 types of compounds: a green derivative, originated by the covalent binding of the heme with the apoprotein, and a dimeric Lb, originated by an intramolecular Tyr-Tyr bond (Moreau et al., 1995).

Leghemoglobins are synthesized by the plants when they are colonized by a symbiotic rhizobium to scavenge the excess of O_2_ which can inhibit the NITROGENASE activity. Phytogbs exist in nodules but also in other organs like roots and leaves of all plants and their concentration range from 100 nM under normal conditions to 5–20 μM when induced by different kind of stresses or hormones (Gupta et al., 2011a). Instead, Lbs are found at millimolar concentrations in the nodules of legumes and are responsible for the typical red color of nodules (Gupta et al., 2011a).

All types of Phytogbs were shown to be highly expressed in *L. japonicus* nodules (Bustos-Sanmamed et al., 2011). Remarkably, LjPhytogb1-1, one protein of the Type 1 family, was proved to have an extremely high affinity for O_2_ (*K* = 0.05 nM) (Sainz et al., 2013). Because of its high O_2_ affinity, this protein remains oxygenated and active even in the presence of CO. This may be important in nodules, where CO can be formed in significant amounts as result of the Lb degradation by heme oxygenases (Baudouin et al., 2004; Sainz et al., 2013). As evidenced in the *L. japonicus* Phytogb1 overexpressing lines (LjPhytogb1-1), these proteins were shown to have a positive effect on the activity of the nodule during the *L. japonicus-M*. *loti* symbiosis and to delay senescence (Fukudome et al., 2018).

In the case of RNS, there is no specific enzymatic systems to scavenge them. Thus, any non-specific system can be relevant to attenuate the deleterious effect of RNS. This is the case of Phytogbs, which can directly scavenge RNS *in vitro* (Herold and Puppo, 2005) and *in vivo* in nodules of soybean and *L. japonicus* (Navascues et al., 2012; Sainz et al., 2015). Interestingly, the *L. japonicus* genes of these Phytogbs were shown to be induced by ⋅NO (Shimoda et al., 2005), suggesting a response to scavenge the excess of ⋅NO. As ⋅NO was shown to be high in the nitrogen fixation zone of nodules, the scavenging of ⋅NO by Lbs was suggested to be key to prevent inactivation of NITROGENASE activity by ⋅NO (Puppo et al., 2013). Because the nitration of Lbs requires low pH 5.5, which is more common in senescent nodules, the protection of Lbs against RNS may be more relevant in senescent nodules. Interestingly, both Lbs and Phytogbs can contribute to the generation of NO_3_^–^ after scavenging ⋅NO and O_2_, with the concomitant oxidation of Hb^2+^O_2_ to Hb^3+^, by the cycle Phytogb-⋅NO mentioned above (Herold and Puppo, 2005; Berger et al., 2019).

### Bacterial Contribution to ROS and RNS Scavenging

In the bacteria, a FLAVOPROTEIN and a SINGLE-DOMAIN HEMOGLOBIN from *B. japonicum* were shown to reduce cell death under the exposure to a ⋅NO-donor, suggesting that these proteins are relevant to detoxify the excess of ⋅NO formed as a by-product of NO_3_^–^ assimilation (Cabrera et al., 2015). In *E. meliloti*, a FLAVOHEMOGLOBIN (HMP) was shown to be one of the most important enzymes to detoxify ⋅NO (Meilhoc et al., 2010; Cam et al., 2012).

Likewise, excessive ROS can be controlled by non-specific scavengers in the rhizobium. For instance, exopolysaccharides are produced in large amounts by *Rhizobium leguminosarum* bv. *trifolii* and play a significant protective role as a barrier against the ROS produced by the clover plants during the symbiotic interaction (Jaszek et al., 2014). Not only the ROS scavenging but also its production is a key factor to determine ROS cellular concentration. As mentioned above, ROS production in the legumes can be also regulated by the rhizobium through nodulation factors (Damiani et al., 2016). Understanding the mechanisms controlling ROS and RNS concentration in the plant-rhizobium interaction has been key to produce and use genetic tools that allow manipulating ROS and RNS levels and unrevealing their importance in this process.

## Signaling Role of ⋅NO in Nitrogen Fixation

Given the evidence that ⋅NO regulates the transcription of genes encoding for different NITROGENASE activity and that it can also modulate NITROGENASE activity at posttranslational level, it is clear that ⋅NO can play a signaling role in nitrogen fixation. The evidence presented here up to now has been focused on NITROGENASE and close related genes. However, we know that signaling molecules interact with signaling cascade pathways involving phytohormones.

**FIGURE 3:**
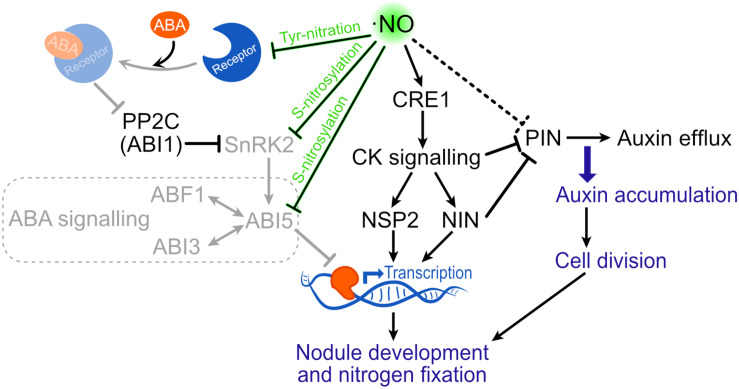
Putative signaling effect of ⋅NO on nodule development. ⋅NO has been shown to induce the transcription of CRE1. Through this way, ⋅NO could promote CK signaling which is known to participate in nodule development. Moreover, ⋅NO is suggested to repress PIN expression resulting in auxin accumulation and cell division. Finally, the binding of ABA to the PYR/PYL/RCAR receptor can be avoided by tyrosine nitration, not allowing the inactivation of PP2C, which inhibits SnRK2 and the downstream ABA signaling through ABI5. ⋅NO is also known to directly inactivate both SnRK2 and ABI5 by *S*-nitrosylation, which also results in the suppression of ABA signaling. By these mechanisms, ⋅NO could attenuate ABA-mediated suppression of nodule development (shown in lower opacity). ABF1, ABA RESPONSIVE ELEMENT-BINDING FACTOR 1; ABI, ABA INSENSITIVE; CK, cytokinin; CRE1, CYTOKININ RESPONSE 1; NIN, NODULE INCEPTION; NSP2, NODULATION SIGNALING PATHWAY 2; PP2C, PROTEIN PHOSPHATASE OF THE TYPE IIC CLASS; SnRK2, SNF1-RELATED PROTEIN KINASE 2.

Although there is a strong body of evidence suggesting a role of ⋅NO in the legume-rhizobia interaction, it is still not clear how ⋅NO promotes this interaction. A potential mechanism includes the activation of CK signaling by ⋅NO. In the *M. truncatula–E. meliloti* symbiosis, the gene encoding for the CK receptor CRE1 (CYTOKININ RESPONSE 1) of this legume, which is the sole receptor mediating CK signaling to induce nodulation (Gonzalez-Rizzo et al., 2006), was observed to be induced by ⋅NO (Ferrarini et al., 2008; del Giudice et al., 2011). Downstream CK signaling, the transcription factors NODULE INCEPTION (NIN) and NODULATION SIGNALING PATHWAY 2 (NSP2) promote nodule development (Schauser et al., 1999; Murakami et al., 2007; Suzaki et al., 2012). Thus, the induction of CRE1 by ⋅NO illustrates a potential mechanism by which the ⋅NO produced soon after the infection could promote the establishment of the symbiosis at early stages (Figure 3).

Auxin accumulation is also important for nodule development, and both flavonoid and CK inhibit AUX transport to promote AUX accumulation in proliferating cortical cells (Suzaki et al., 2012). Based on evidence showing that ⋅NO down-regulate the expression of PIN AUX efflux carriers genes in arabidopsis and rice, Berger et al. (2019) suggested that ⋅NO can also contribute to the control of AUX transport by repressing the expression of PIN proteins to produce AUX accumulation and ultimately cell division in the nodule (Figure 3). Yet, it is not clear the mechanism by which ⋅NO can regulate PIN levels. A potential mechanism can be through the ⋅NO-induced CK signaling mentioned above (via CRE 1). CK was shown to control the PIN proteins and its degradation to redirect AUX efflux and establish local AUX accumulation (Plet et al., 2011; Marhavý et al., 2014). Through this mechanism, the ⋅NO-induced CK signaling could contribute to reducing PIN levels (Figure 3). In future research, it would be interesting to evaluate at protein level if CRE1 is induced by ⋅NO.

Abscisic acid (ABA) plays an important role in plant development and has been shown to negatively affect nodule development at different stages in several legumes (Phillips, 1971; Suzuki et al., 2004; Tominaga et al., 2009, 2010; Nagata and Suzuki, 2014). For instance, in the *L. japonicus* ABA-insensitive mutant *enf1* (enhanced nitrogen fixation 1), both the nitrogen fixation and the number of nodules formed were almost double (Tominaga et al., 2009), suggesting that ABA inhibits nodule formation and nitrogen fixation. Accordingly, when *L. japonicus* WT plants were treated with an inhibitor of ABA synthesis, the number of nodule and nitrogen fixation activity was increased (Tominaga et al., 2010). This negative effect of ABA was related to its capacity to inhibit CK signaling, isoflavonoid synthesis and the calcium spiking produced after Nod-factor perception (Miwa et al., 2006; Ding et al., 2008; Tominaga et al., 2010; Nagata and Suzuki, 2014). *M. truncatula* lines over expressing the *A. thaliana abi1-1* allele, which codifies for the PROTEIN PHOSPHATASE OF THE TYPE IIC CLASS (PP2C) that is able to inhibit ABA signaling (Figure 3), showed hypernodulation phenotype (Ding et al., 2008). In this line, ABA was shown to suppress Nod-factor signal transduction and CK induction (Ding et al., 2008). The inhibition of ABA signaling by PP2C is through its capacity to dephosphorylate and inactivate the serine/threonine kinases SnRK2. In a similar way, ⋅NO was shown to be able to inactivate some family members of the SnRK2 kinases by *S*-nitrosylation (Wang et al., 2015a, b). Moreover, ⋅NO was shown to negatively regulate ABA signaling up- and down-stream SnrK2 (Figure 3; Albertos et al., 2015; Castillo et al., 2015; Wang et al., 2015a). Considering that ⋅NO production is enhanced during the legume-rhizobia interaction (Figure 1), it is expected that in such situation the ABA signaling pathway will be more prone to be affected by ⋅NO (Figure 3).

Besides affecting the response of hormones directly involved in nodule development (i.e., CK and AUX), ⋅NO also modulates the response to salicylic acid (SA) (Klessig et al., 2000) and jasmonic acid (JA) (Huang et al., 2004), two hormones known to be involved in the innate immune response of plants (Betsuyaku et al., 2018; Tarkowski et al., 2020). Thus, it has been considered that by modulating the plant innate immune response ⋅NO could potentially modulate the establishment of the symbiosis (Tartaglia et al., 2019). In *M. truncatula* and *L. japonicus*, the SA-mediated plant defense pathways were shown to inhibit the formation of determinate- and indeterminate-type of nodules (Stacey et al., 2006). Regarding JA however, there is no clear evidence that it can modulate nodule development, in fact, transgenic plants over-expressing and silencing an enzyme of the JA biosynthesis (ALLENE OXIDE CYCLASE) were unable to affect development and function of nodules (Zdyb et al., 2011). It is not surprising that ⋅NO had a differential effect on SA and JA signaling, as SA and JA usually have a mutually antagonist effect (Betsuyaku et al., 2018). Further research would contribute to understand the importance of the innate immune response of plants in the legume-rhizobia interaction and how relevant ⋅NO is to this process.

## Perspectives

### The Effects of ⋅NO on Nitrogen Fixation

Different reports have suggested that ⋅NO can both promote and reduce nitrogen fixation. Although the conclusions in some cases are opposite, the results are not necessarily opposite. Furthermore, the effect of ⋅NO on nitrogen fixation depends on whether it acts directly on the NITROGENASE, or on upstream effectors, the concentration of ⋅NO and the time of the exposure. In this section, we will refer first to the evidence concluding a positive effect of ⋅NO on nitrogen fixation, followed by those suggesting a negative effect.

#### Positive Effects of ⋅NO on Nitrogen Fixation

As presented above in section “⋅NO in Legume-Bacteria Interaction”, different works have concluded that ⋅NO is necessary for the correct establishment of the legume-rhizobia interaction and nodule development. This would ultimately result in better nitrogen fixation and thus can be considered as evidence supporting a positive effect of ⋅NO on BNF. Here, we discussed the potential mechanisms by which ⋅NO can have this effect, involving CK and AUX signaling pathways (Figure 3). Even before the legume-rhizobia interaction takes place, greater ⋅NO levels were suggested to repress plant defense reactions which in turn would promote the correct infection and nodule establishment (Berger et al., 2019). This would be another mechanism by which ⋅NO promotes BNF.

**FIGURE 4:**
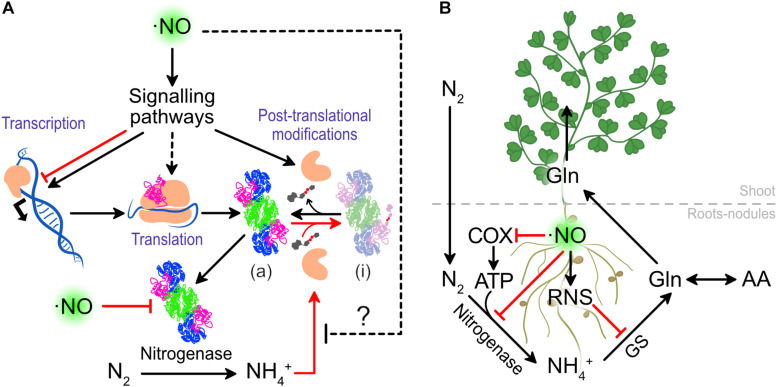
The effect of ⋅NO on nitrogen fixation and assimilation. **(A)** Effects of ⋅NO on NITROGENASE activity. **(B)** Detrimental effects of ⋅NO and RNS on nitrogen fixation and assimilation. In red are presented the interactions that lead to a lower nitrogen fixation and assimilation. COX, CYTOCHROME OXIDASE; GS, GLUTAMINE SYNTHASE.

Nitric oxide levels can also modulate the NITROGENASE activity and in this way directly affect nitrogen fixation. Treatments of 0.1 mM sodium nitroprusside (SNP, ⋅NO donor) increased the NITROGENASE activity from *L. japonicus* nodules after 27 h by an unknown mechanism (Kato et al., 2010). As it is widely known that ⋅NO directly inhibits the NITROGENASE activity, we interpret these results as a positive effect on upstream effectors, that results in a greater NITROGENASE expression or activity (Figure 4A). Understanding this mechanism can shed light on key molecules controlling NITROGENASE activity.

#### Negative Effects of ⋅NO on Nitrogen Fixation

Some reports have suggested that ⋅NO can difficult the establishment of the legume-rhizobia interaction. For instance, *L. japonicus* plants treated with ⋅NO donors and lines having higher ⋅NO levels, due to a mutation in the *Phytogb1* gene, were shown to have problems to form the infection threat (Fukudome et al., 2016). Moreover, they observed lower number of nodule in the lines having increased ⋅NO levels (Fukudome et al., 2016). At first sight, this would be contradictory than other reports showing a positive effect of the ⋅NO in the infection thread formation (Pii et al., 2007; del Giudice et al., 2011), but it may mean that altering the equilibrium of ⋅NO, either to more or less, always results in negative effect on the infection thread formation, which in some cases was interpreted as ⋅NO promoting the process and in other as inhibiting it.

The clearest negative effect of ⋅NO on nitrogen fixation is its capacity to inhibit NITROGENASE activity. The same authors who demonstrated in *L. japonicus* that ⋅NO enhances NITROGENASE activity when the nodules are treated with the 0.1 mM SNP, also showed that at higher concentrations (at 1 mM or above) SNP inhibits its activity (Kato et al., 2010). This was previously observed *in vitro* using NITROGENASE isolated from bacteroids of soybean nodules (Trinchant and Rigaud, 1982). This *in vitro* evidence demonstrates that ⋅NO can directly interact and inhibit NITROGENASE activity (Figure 4), independently of signaling events acting up-stream.

Besides the direct effect of ⋅NO on NITROGENASE, ⋅NO was also suggested to attenuate NITROGENASE activity at the transcriptional level in *B. japonicum* (Sánchez et al., 2010). In particular, nodules of soybean subjected to flooding increased the ⋅NO content due to a higher periplasmic NR (Nap) activity, and in such situation, a down-regulation of the expression of *B. japonicum nifH* gene, which encodes the Fe subunit of the NITROGENASE, was observed (Sánchez et al., 2010).

Because plant Hbs and bacterial HMP were shown to be important ⋅NO-detoxifying proteins in plants and rhizobia, respectively, different works used mutants and/or overexpressing lines for these proteins to control the endogenous levels of ⋅NO. From the plant side, Hbs overexpression in *L. japonicus* resulted in lower ⋅NO levels, a greater number of formed nodules and higher NITROGENASE activity, suggesting that basal levels of ⋅NO inhibit nitrogen fixation (Shimoda et al., 2009). From the bacterial side, Cam et al. (2012) used *E. meliloti Hmp* null mutant strain and *Hmp* overexpressing strain to infect *M. truncatula* nodules and manipulate the levels of ⋅NO. Through this approach, they showed that the *Hmp* null mutant had greater ⋅NO levels and a dramatic decrease in the NITROGENASE activity (Cam et al., 2012). They also found that nodule senescence was faster and greater in the *Hmp* null mutant, but smaller in the *Hmp* overexpressing strain (Cam et al., 2012). In this way, Cam et al. (2012) showed that keeping ⋅NO under control is not only important to protect NITROGENASE activity, but also to delay nodule senescence which ultimately will result in extended nitrogen fixation by the plant.

Together, the current evidence demonstrates how ⋅NO can regulate NITROGENASE activity not only at the transcriptional level but also at post-translational level (Figure 4A), and both the plants and the rhizobia play an important role controlling ⋅NO homeostasis.

#### The Effects of ⋅NO and RNS on Nitrogen Fixation and Assimilation

Beyond the effect of ⋅NO on NITROGENASE, ⋅NO is known to inhibit CYTOCHROME OXIDASE in different organisms including plants (Millar and Day, 1996). Thus, at high levels, ⋅NO can threat the ATP production which is necessary for large quantities for NITROGENASE activity (Figure 4B). In such a situation, the ALTERNATIVE OXIDASE (AOX) of nodules was suggested to play a role to allow nodule respiration (Millar et al., 1997), although ATP production would be still minimized.

Once the nitrogen is reduced to ammonium by the rhizobia, the plants assimilate the ammonium to transport the nitrogen in an organic form and reduce the levels of free ammonium which is toxic at high concentrations. The GLUTAMINE SYNTHETASE-GLUTAMATE SYNTHASE (GS-GOGAT) plays a major role in the assimilation of N to amino acids. The GLUTAMINE SYNTHASE 1 of legumes was shown to be nitrated, both in roots of *L. japonicus* under drought stress (Signorelli et al., 2019) and in nodules of *M. truncatula* (Melo et al., 2011), causing the inactivation of the enzyme (Melo et al., 2011). As protein nitration requires ⋅NO and ROS, we can assume that uncontrolled ⋅NO would not only inhibit NITROGENASE and cytochrome oxidase activity but also GLUTAMINE SYNTHETASE, compromising the total capacity of the legumes to reduce and assimilate atmospheric nitrogen (Figure 4B).

#### Our Conclusions on the Effect of ⋅NO on Nitrogen Fixation and Assimilation

Taking all together, the current evidence suggests that the positive effect of ⋅NO on nitrogen fixation is associated to the promotion of legume-rhizobia interaction and signaling pathways controlling the NITROGENASE activity (Figures 1, 3, 4A). On the other hand, the negative effects of ⋅NO on nitrogen fixation and assimilation seem to be mostly related to the direct inactivation of the main actors in the process of nitrogen fixation and assimilation by ⋅NO and RNS (Figure 4). Therefore, most of the evidence showing a negative effect of ⋅NO on BNF relay on the direct action of ⋅NO (or RNS) in a one to one ratio (e.g., CYTOCHOROME OXIDASE and NITROGENASE inactivation), whereas those linked to a positive effect seem to act on signaling pathways (e.g., CK, AUX, ABA) having an amplifier effect. Therefore, we speculate that, at physiological conditions, ⋅NO is more likely to have a positive effect on BNF.

Both plants and rhizobia ⋅NO-detoxifying systems were observed to be essential to control ⋅NO at basal levels and avoid NITROGENASE inactivation. The induction of early nodule senescence observed in different reports suggests that an early and uncontrolled increase of ⋅NO in the nodule can present a threat for plant productivity. In the experiments mentioned in this review, the levels of ⋅NO were manipulated to be abnormal, either using ⋅NO donors, mutant plants or rhizobium strains. However, we know that in wild type plants, levels of ⋅NO can be triggered by environmental factors. Therefore, it seems to be important to understand how to prevent the spikes of ⋅NO that can threat the biological fixation and nodule viability.

### Alternative Signaling Pathways by Which ⋅NO Could Modulate BNF

The N-end rule pathway of proteolysis (NERP) is a mechanism by which O_2_, and also ⋅NO, can act as signaling molecules to promote protein degradation. In particular in plants, some ERF were shown to be subjected to proteasomal degradation under normoxia through this pathway (Gibbs et al., 2014; Meitha et al., 2018). As the nodule also has a low O_2_ environment, ERF would be stable in such organ becoming a potential molecular switch that can be regulated by ⋅NO via the NERP. Therefore, evaluating the involvement of ERF in nodule seems to be a worth exploring area in the future.

Likewise, most research about ABA signaling has been focused on the control of dormancy in seeds or, at vegetative level, on stress responses, thus it is not clear yet which transcriptions factors could be involved downstream SnRK2 in nodulation. The basic leucine zipper transcription factor ABSCISIC ACID INSENSITIVE 5 (ABI5) is suggested to be the integrator of ABA and other phytohormone signaling during stress conditions and developmental processes (Skubacz et al., 2016). For example, CK is known to negatively regulate ABI5 protein level (Guan et al., 2014). Therefore, it would not be surprising that ABI5 also controls nodule formation. Currently, the role of ABI5 in nodule development is largely unexplored and further research in this direction would contribute to a better understanding of the molecular mechanism underpinning nodule development by phytohormones.

Finally, most of our knowledge about NITROGENASE regulation is limited to the transcriptional regulation of *nif* genes. However, in many nitrogen-fixing bacteria, the NITROGENASE is known to be regulated by post-translational modifications (Pope et al., 1985; Huergo et al., 2009; Heiniger et al., 2012). In particular, in the presence of ammonium, an ADP-ribosyltransfease (DraT2) transfers an ADP-ribose to a conserved arginine on DINITROGENASE REDUCTASE (NifH) to inactivate the NITROGENASE activity (Heiniger et al., 2012). This post-translational regulation involves several proteins such as NtrB, NtrC, GlnK2, DraG, which if inactivated by ⋅NO would promote the NITROGENASE activity by interfering with the inactivation of NITROGENASE in presence of ammonium (Figure 4A). Therefore, this seems worth exploring because understanding the effect of ⋅NO on these proteins could led to potential mechanisms by which ⋅NO could modulate the post-translational inhibition of the NITROGENASE activity.

### What Is the Role of Abiotic Stress-Induced ⋅NO on BNF?

The establishment of the nodule and BNF are known to be extremely sensitive to modest drought conditions in many species (Serraj et al., 1999; Sinclair and Serraj, 1995). Because of that, there have been many reports focused on how drought and other environmental factors affect BNF. However, there are virtually no studies about the effect of endogenous abiotic stress-induced ⋅NO on BNF. It would be interesting to evaluate this because most environmental stresses result in the overproduction of ⋅NO and nitrosative stress. For instance, in the legumes *L. japonicus* and *Pisum sativum* an overproduction of ⋅NO in roots was reported when they are exposed to environmental stresses (Signorelli et al., 2013; Lehotai et al., 2016). However, in these studies, the plants were grown in a medium having high levels of nitrate (NO_3_^–^), which can be used by NR and contribute to ⋅NO overproduction. This does not represent a situation in which nodulation would take place, as it requires low levels of N in the soil, and perhaps in such conditions, there is no ⋅NO overproduction. Therefore, it would be important to evaluate if there is ⋅NO overproduction in nodulation and stress conditions, and what the effects of scavenging this ⋅NO are.

## Conclusion

The involvement of ⋅NO throughout the whole process of legume-rhizobia interaction has been well documented, and most reports agree that ⋅NO is necessary for the correct establishment of the interaction. Both the bacteria and the plants have been shown to contribute to ⋅NO production and scavenging, and some findings points to the possibility that ⋅NO could promote the nodule formation by enhancing CK signaling pathway and interfere with AUX and ABA signaling pathways. Moreover, transcriptomic analyses have suggested that its metabolism is more active during nodule maturation. In mature nodules, many proteins have been identified as S-nitrosylated, including the thiol peroxidase GPX1 which is key for H_2_O_2_ sensing and transmission of oxidative signals. It is likely that this type of post-translational modification is the most responsible for the regulatory role of ⋅NO. To counteract this modification, legume plants possess different hemoglobins which play a significant role in ROS and RNS metabolism by contributing to ROS production and ⋅NO scavenging. In the near future, it is likely that we expand our knowledge about enzymes that are S-nitrosylated *in vivo* and the effect of such modification.

## Author Contributions

SS conceived the idea and structure of the manuscript, wrote the first draft of the manuscript, and illustrated all the figures. ST-D contributed to the section of metabolism of ⋅NO in nodules. MS contributed to the writing of the section about ROS and RNS homeostasis in the nodule and designed Figure 2. JM revised the whole manuscript.

## Conflict of Interest

The authors declare that the research was conducted in the absence of any commercial or financial relationships that could be construed as a potential conflict of interest. The handling editor declared a past co-authorship with several of the authors SS and JM.
